# Prosthetic energy return during walking increases after 3 weeks of adaptation to a new device

**DOI:** 10.1186/s12984-018-0347-1

**Published:** 2018-01-27

**Authors:** Samuel F. Ray, Shane R. Wurdeman, Kota Z. Takahashi

**Affiliations:** 1 Department of Biomechanics, University of Nebraska at Omaha, Omaha, NE USA; 2 Department of Clinical and Scientific Affairs, Hanger Clinic, Houston Medical Center, Houston, TX USA

**Keywords:** Amputation, Mechanical work, Gait, Biomechanics

## Abstract

**Background:**

There are many studies that have investigated biomechanical differences among prosthetic feet, but not changes due to adaptation over time. There is a need for objective measures to quantify the process of adaptation for individuals with a transtibial amputation. Mechanical power and work profiles are a primary focus for modern energy-storage-and-return type prostheses, which strive to increase energy return from the prosthesis. The amount of energy a prosthesis stores and returns (i.e., negative and positive work) during stance is directly influenced by the user’s loading strategy, which may be sensitive to alterations during the course of an adaptation period. The purpose of this study was to examine changes in lower limb mechanical work profiles during walking following a three-week adaptation to a new prosthesis.

**Methods:**

A retrospective analysis was performed on 22 individuals with a unilateral transtibial amputation. Individuals were given a new prosthesis at their current mobility level (K3 or above) and wore it for three weeks. Kinematic and kinetic measures were recorded from overground walking at 0, 1.5, and 3 weeks into the adaptation period at a self-selected pace. Positive and negative work done by the prosthesis and sound ankle-foot were calculated using a unified deformable segment model and a six-degrees-of-freedom model for the knee and hip.

**Results:**

Positive work from the prosthesis ankle-foot increased by 6.1% and sound ankle-foot by 5.7% after 3 weeks (*p* = 0.041, 0.036). No significant changes were seen in negative work from prosthesis or sound ankle-foot (*p* = 0.115, 0.192). There was also a 4.1% increase in self-selected walking speed after 3 weeks (*p* = 0.038). Our data exhibited large inter-subject variations, in which some individuals followed group trends in work profiles while others had opposite trends in outcome variables.

**Conclusions:**

After a 3-week adaptation, 14 out of 22 individuals with a transtibial amputation increased energy return from the prosthesis. Such findings could indicate that individuals may better utilize the spring-like function of the prosthesis after an adaptation period.

**Electronic supplementary material:**

The online version of this article (10.1186/s12984-018-0347-1) contains supplementary material, which is available to authorized users.

## Background

After undergoing a lower limb amputation, individuals must relearn to walk with a prosthesis that replaces anatomical foot and ankle structures. These individuals often walk with lower speeds and greater metabolic cost, in comparison to control populations [1–3]. This discrepancy has been lessened, but not eliminated, with the advent of modern energy-storage-and-return (ESR) prosthetic feet [4–6]. These individuals have a tendency to exert greater forces on the sound limb [7], and such over-reliance may contribute to secondary problems, such as lower back pain, osteoporosis, and osteoarthritis of the contralateral knee and hip [7, 8].

Since prosthetic structures must replace basic functions of an anatomical system, such as supplying forward propulsion and upright support [9, 10], the way in which a person utilizes and interacts with a prosthesis may be a key factor in regaining walking ability. Many contemporary studies either analyze prosthesis users in comparison to control subjects [11–14], or compare gait outcomes among various prosthetic devices [15–20]. However, few studies have analyzed adaptation to a prosthetic device over a prolonged period [21–23]. Here, we refer to ‘adaptation’ as changes seen over a multi-week timespan in gait outcomes of individuals with amputation.

A potentially valuable approach for understanding prosthetic adaptation is the analysis of mechanical power and work. Modern designs of prostheses attempt to maximize positive work output of the devices [24], by either passive-elastic, active [12], or processor-controlled [25] components in efforts to mimic the power generation performed by the sound foot and ankle during gait. Prior research including passive-elastic [11] and powered prostheses [12] has established the importance of positive work generation during push-off; in particular, its role in increasing self-selected walking speed [12], decreasing metabolic energy expenditure [12, 26], and promoting gait symmetry and balance [11, 27].

For an unpowered ESR prosthesis, the amount of negative and positive work of the prosthesis – equivalent to energy storage/dissipation and return, respectively – is directly influenced by the material properties and geometry of passive ESR feet [28–30]. In addition, mechanical work profiles may be directly related to how a user loads and unloads the prosthetic limb, offering unique insights into a user’s interaction with a prosthesis. It is currently unclear how energy storage and return characteristics may change over the course of adaptation. In this context, assuming that the mechanical properties of the prosthetic ankle-foot components have remained the same, any change in prosthetic mechanical work should reflect changes in loading patterns initiated by the individual.

The purpose of our study was to quantify adaptation to a new ESR prosthesis through analyzing mechanical work profiles during walking at the ankle-foot, knee, and hip level over a 3-week period. We hypothesized that following a 3-week adaptation to a new device, the prosthesis negative and positive work would increase, indicative of greater use of the spring-like behavior of the ESR device. With increased push-off from the prosthesis, we also hypothesized that positive work output of the sound limb structures (ankle-foot, knee, and hip) would decrease. This may reflect a lessened dependence on the sound limb for locomotion, which may be in line with the goals of gait training interventions to improve limb symmetry [27, 31].

## Methods

### Participants

Twenty-two individuals with unilateral, transtibial-level amputation were retrospectively analyzed from a previous study [21]. Cause of amputation included traumatic (*n* = 14), vascular (*n* = 5), and other (*n* = 3). Subject group demographics are summarized in Table 1. All participants were indicated as Medicare Functional Classification Level K3 or K4 ambulators, and had their current prescribed prosthesis longer than 30 days.

**Table 1:** Subject-level Demographics

Subject	Age (yrs)	Height (cm)	Mass (kg)	Years since Amputation	Original Prosthesis	Prescribed Prosthesis
1	42	184.6	125.0	3	OSR Ceterus	FI Renegade
2	66	176.5	121.6	8	CPI Soleus	OSR Variflex
3	57	186.3	98.6	1	OSR Variflex Evo	WW Fusion
4	55	163.2	64.1	10	OB Trias	FI Senator
5	58	192.7	115.2	6	OSR Variflex	WW Fusion
6	61	189.0	108.4	7	OSR Ceterus	WW Duralite
7	63	178.0	108.4	5	OSR Ceterus	FI Renegade Torsion
8	76	172.3	92.3	5	EL Echelon	WW Fusion
9	62	179.5	86.5	14	OSR Ceterus	WW Duralite
10	65	188.0	100.5	8	CPI Soleus	FI Senator
11	53	174.5	121.8	2	OSR Talux	OSR Variflex
12	33	169.1	66.2	10	OSR Variflex	AD Rush foot
13	58	169.0	98.0	2	OSR Flexfoot Assure	AD Rush foot
14	50	188.7	93.0	11	FI Sierra	FI Renegade
15	47	172.0	113.9	8	CPI Trustep	FI Pacifica
16	45	166.0	121.8	3	OSR Variflex LP	FI Pacifica
17	44	182.7	87.3	24	OSR Ceterus	AD Rush foot
18	41	173.8	93.4	20	CPI Trustep	WW Fusion
19	45	169.2	122.5	4	FI Renegade	FI Senator
20	53	178.8	133.8	2	OB Axtion	FI Renegade
21	66	177.4	80.7	0.6	OSR Sure-flex	EL Elite
22	27	173.8	87.3	11	CPI Soleus	FI Renegade
Mean (±SD)	53.0 (±11.8)	177.5 (±8.2)	101.8 (±19.1)	7.5 (±6.0)		

All participants were indicated as Medicare Functional Classification Level K3 or K4 ambulators, and had their original prosthesis longer than 30 days. Prosthetic manufacturer *abbreviations: AD* Ability Dynamics, *CPI* College Park Industries, *EL* Endolite, *FI* Freedom Innovations, *OB* Ottobock, *OSR* Össur, *WW* Willowwood.

### Procedures

**Fig. 1:**
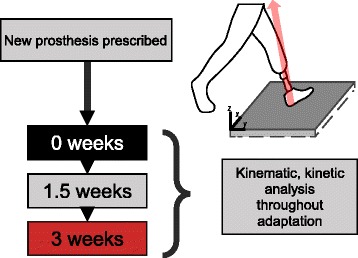
Experimental Protocol. *N* = 22 subjects were prescribed a new prosthetic foot at their current functional level by a certified prosthetist. Fitting and alignment were performed on the subject’s first visit to the gait laboratory, after which the first data collection was performed (0 weeks). Subjects then wore the prosthesis at home and in the community, and came back for data collections after 1.5 weeks and 3 weeks. Each collection consisted of kinetic and kinematic data from overground walking trials at a self-selected speed. Subjects were retrospectively analyzed from a previous study conducted [21].

Participants in the study were given a new, passive energy storage and return-type prosthetic foot to wear for the duration of the 3 week adaptation period (Fig. 1). The new prosthesis was based on the participants’ Medicare Functional Classification Level at the time of testing. Participants used their same socket and suspension system as their regular prosthesis, and were issued a new foot customized by their body mass, height, and foot size. During their initial visit, participants received a new prosthetic foot and were aligned by a certified prosthetist on-site. The alignment prior to the initial gait analysis occurred consistent with clinical alignment process in less than 10 min. Once the prosthesis was properly aligned, initial gait analysis (0 weeks adaptation) was performed. After the first data collection, participants wore their new prosthesis home and returned to the lab after 1.5 weeks for second data collection. Participants then wore the new prosthesis for 1.5 more weeks and returned again for a final data collection (3 weeks adaptation).

During each data collection, participants walked overground on an embedded force plate (Kistler, Amherst, NY) at a self-selected speed. Kinetic data were captured from the force plate at 600 Hz, while kinematic data were captured by a 12-camera motion capture system at 60 Hz (Motion Analysis Corp., Santa Rosa, CA). Kinetic and kinematic data were low-pass filtered at cutoff frequencies of 11 Hz and 7 Hz, respectively. The cutoff frequencies were determined using residual analysis [32]. Twenty seven reflective markers were placed on various anatomical locations on the lower limbs to allow three dimensional relative joint angle calculations. On the prosthetic limb, markers were placed on analogous locations as the sound limb. A minimum of 5 clean force plate steps were recorded from each leg from each participant for analysis. A clean step was defined as full foot contact with only one force plate.

### Analysis

A unified deformable (UD) segment analysis [33] was used to quantify mechanical power profiles of prostheses and sound ankle-foot structures. While traditional analysis (e.g., inverse dynamics-based methods) rely on rigid body assumptions, the UD analysis accounts for the power and work contributions from deforming structures. The analysis also does not require a joint definition. Thus, the UD analysis is well suited to study prostheses that may not have a true ankle joint articulation, facilitating direct comparisons between anatomical ankle-foot structures and various prosthetic designs [34]. The analysis defines a ‘unified’ segment, in which the shank is assumed to be rigid, and everything distal to the shank is deformable. Deformation of distal components is quantified by the translational velocity ($\overrightarrow{v}$_*cop*_), which is the velocity of a point on the unified segment as it coincides with location of the center-of-pressure (Eq. 1). $\overrightarrow{v}$_*cop*_ is found using Eq. 1, where $\overrightarrow{v}$_*cm*_ is the translational velocity of the shank’s center of mass, $\overrightarrow{\omega}$ is the rotational velocity of the shank, and $\overrightarrow{r}$_*cop*_ is the displacement of the center of pressure relative to the shank’s center of mass.

1 $$ {\overrightarrow{v}}_{cop}={\overrightarrow{v}}_{cm}+\left(\overrightarrow{\omega}\ x\ {\overrightarrow{r}}_{cop}\right) $$

Then, the dot product is taken between $\overrightarrow{v}$_cop_ and the ground reaction force ($\overrightarrow{F}$_grf_) and summed with the dot product of free moment ($\overrightarrow{M}$_*free*_) and ω to calculate power (P_UD_) (Eq. 2).

2 $$ {P}_{UD}={\overrightarrow{F}}_{grf}\bullet {\overrightarrow{v}}_{cop}+{\overrightarrow{M}}_{free}\bullet \overrightarrow{\omega} $$

We note that all of the denoted vectors (Eqs. 1 and 2) are three-dimensional vectors, with exception of ${\overrightarrow{M}}_{free}$, which only acts normal to the ground surface. Integrating *P*_*UD*_ over time quantified total energy (work) of the prosthesis over a gait cycle due to elastic energy storage and return. When this technique was applied to the sound side, it quantified energy profiles of the sound ankle-foot system as a whole.

Power and work profiles at the knee and hip joints were quantified by using a six-degrees-of-freedom technique [35, 36]. This technique quantifies the summed effect of all structures surrounding a joint, including power contributions from rotational and translational movement.

A one-way repeated measures ANOVA was used to determine the effect of time (visit) on various outcome variables, which included total positive work output by the prosthetic and sound-side ankle-foot, knee, and hip, as well as the total negative work done by the prosthetic and sound-side ankle-foot, knee, and hip.

## Results

**Fig. 2:**
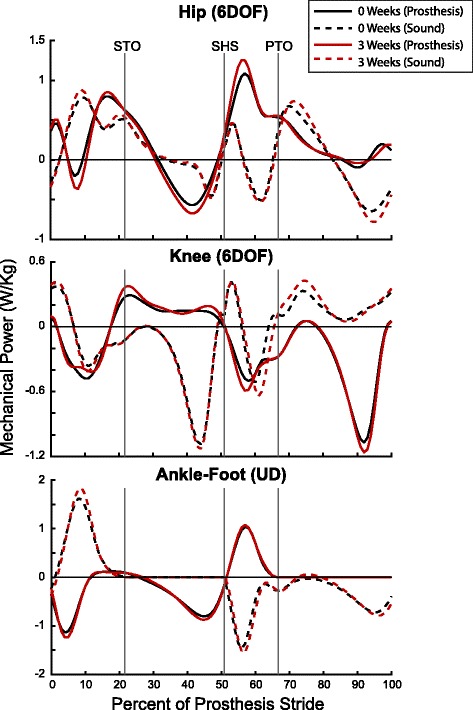
Mechanical power of lower extremity over gait cycle. Ensemble mean mechanical power curves are shown for the prosthetic foot (solid lines) and sound ankle-foot system (dashed lines). Curves are plotted as a percentage of prosthetic limb stride, with sound limb heel strike happening at approximately 51%. Mechanical power at the ankle-foot was calculated using a Unified Deformable segment model, and eventually integrated to calculate mechanical work. Knee and hip powers were calculated using a six-degree-of-freedom power analysis. Vertical lines indicate gait events: STO = Sound limb Toe-Off, SHS = Sound limb Heel Strike, PTO = Prosthetic limb Toe-Off

The mechanical power profiles of the prosthetic ankle-foot structures showed evidence of energy storage and return (Fig. 2). Immediately following heel strike, the prostheses showed negative power, exhibiting either energy storage or dissipation. During mid-stance, the prostheses again showed negative power. During terminal stance, the prostheses exhibited positive power – indicative of energy return during push-off.

**Fig. 3:**
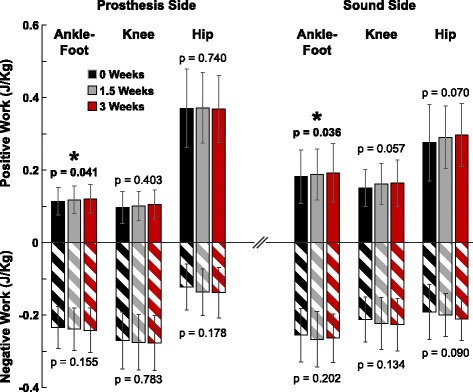
Mechanical work changes over 3 week period. Group mean (± 1 SD) joint-level positive and negative work values are shown for 0 weeks (black), 1.5 weeks (gray), and 3 weeks (red) of adaptation to a new prosthetic device. * = denotes significance as revealed by one-way repeated measures ANOVA for effect of time on mechanical work (*p* < 0.05). Prosthetic ankle-foot positive work (i.e., energy return) increased after adaptation (*p* = 0.041), including a 6.1% increase at 3 weeks compared to 0 weeks. Sound limb ankle-foot positive work also increased after adaptation (*p* = 0.036), including a 5.7% increase at 3 weeks compared to 0 weeks. No other work changes were statistically significant. All passive energy-storage-and-return feet did not and cannot exceed 100% energy return, but an increase in positive work generation was observed without a corresponding increase in negative work, contrary to our hypothesis

Following a three-week adaptation, the prosthesis positive work increased (*p* = 0.041), including a 6.1% increase at week 3 compared to week 0 (0.114 J/kg vs 0.121 J/kg) (Fig. 3). There was no significant change in prosthesis negative work (*p* = 0.155). On the sound ankle-foot, there was a significant increase in positive work (*p* = 0.036), including a 5.7% increase at week 3 compared to week 0 (0.182 J/kg vs 0.193 J/kg). There were no significant changes in sound ankle-foot negative work (*p* = 0.202). There was no significant effect of adaptation period on all other joint work profiles, including knee and hip positive and negative work.

## Discussion

The purpose of this study was to quantify adaptation to a new prosthesis through analysis of mechanical work profiles. Assuming that the prosthesis does not change its mechanical properties during the 3-week adaptation period, any changes in prosthetic mechanical work profiles should be directly related to how a user loads and unloads the prosthetic limbs. These changes may reveal unique insights regarding the individual’s interaction with the prosthesis. In line with our hypothesis, prosthesis positive work (i.e., energy return) increased after 3 weeks of adaptation. From a mechanical perspective, these changes in prosthetic energy return could be a beneficial change, as prosthetic ankle-foot structures commonly have reduced push-off in comparison to sound limbs, which may be related to gait asymmetry [11] or increased metabolic cost of transport [12]. Increasing prosthetic energy return is also in line with the design goals of energy-storage-and-return (ESR) feet [1, 4], which seek to increase push-off from the prosthesis side. It should be noted, however, that positive work values are still well below sound limb levels – after 3 weeks, positive work from the prosthesis was only 62.8% of the sound ankle-foot.

**Fig. 4:**
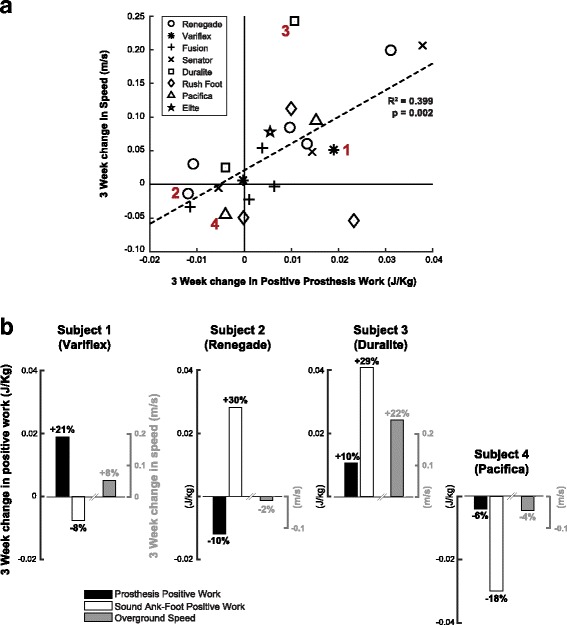
Regression analysis and inter-subject variations. **a:** A significant linear regression (R^2^ = 0.399, *p* = 0.002) was found between change in positive prosthesis work and change in self-selected speed. Changes in speed can be partially explained by changes in prosthesis positive work, but the two may not be entirely interdependent. Furthermore, there was a large amount of inter-subject variation in our results, as four highlighted subjects show. **b:** Subjects 1-4 on **a** are plotted again on **b**, showing each subject’s prescribed prosthesis, as well as changes in prosthesis positive work, sound ankle-foot positive work, and self-selected speed after 3 weeks adaptation. Percentage changes for each variable are shown above and below the corresponding bar graph. Some subjects increased prosthesis work while decreasing sound limb work as hypothesized (subject 1), while others increased sound limb work while decreasing prosthesis work (subject 2). This strategy may indicate that a patient is growing too reliant on their sound limb for locomotion. Some subjects increased sound limb work disproportionately to prosthesis work (subject 3), and even others decreased all variables (subject 4), which may indicate nonresponse to a newly-given prosthetic device

One potential explanation behind the increased prosthetic energy return (following the 3 week adaptation) is an increase in self-selected walking speed. In this study, we did not control for the overground walking speed across the three testing sessions, and we found a 4.1% increase (*p* = 0.038) in self-selected speed after 3 weeks. Furthermore, a significant linear relationship (R^2^ = 0.399, *p* = 0.002) was found between 3-week changes in positive prosthesis work and 3-week changes in speed (Fig. 4a). As ankle-foot work profiles are sensitive to increases in walking speed [37], it is currently unclear whether the increase in prosthetic energy return was due to the process of adaptation or due to the increase in self-selected speed. From regression analysis, we can reason that changes in gait speed could be partially, but not wholly explained by changes in prosthesis work. Furthermore, it is currently difficult to assess whether an increase in prosthetic energy returned caused an increase in self-selected speed, or vice versa. Future controlled experiments may be needed to parse out the influence of walking speed and prosthetic energy return over the course of a prolonged adaptation period.

Since passive ESR prostheses can only return a portion of mechanical energy that they store, we had hypothesized that an increase in prosthetic positive work would coincide with an increase in negative work. Magnitude of negative work of the prosthesis increased by 3.5% over the 3-week period, however, this difference was not significant (*p* = 0.155) and thus did not support our hypothesis. This is not to say that the prostheses generated more positive work than they absorbed. Rather, there was a greater percentage of the negative work that was returned by the prosthesis. For example, the work-ratio (positive work/negative work) slightly increased from 0.482 at Week 0 to 0.495 at Week 3, though this difference was not statistically significant (*p* = 0.193, see Additional file 1). One possible way to increase prosthetic energy may be to change the loading regions of a prosthesis. For example, individuals could have loaded less on the dissipative heel of the prosthesis and more on the flexible keel made to return energy. Due to the nature of our calculations, energy storage and dissipation are both quantified as negative work, and further experiments and analyses may be needed to examine the localized mechanical work distributions within prosthetic foot subregions.

Alongside the increased energy return from the prosthetic ankle-foot structures following a 3-week adaption, we had hypothesized that the positive work production from the sound limb (either ankle-foot, knee, or hip) would decrease (i.e., a more symmetrical work distribution). While there was no change in knee or hip work, the sound ankle-foot positive work increased (Fig. 3), thus rejecting our hypothesis.

While there was a significant group mean increase in prosthesis and sound limb ankle-foot work, a subject-specific analysis revealed highly variable mechanical work and self-selected speed profiles following adaptation. Such subject-specific analyses may offer valuable information of how an individual is integrating a new ESR prosthesis. As an example, data for 4 subjects is shown highlighting the variable patterns. Subject 1 in Fig. 4b showed an increase in prosthesis work with a decrease in sound limb work, and subject 2 showed a decrease in prosthesis work with an increase in sound limb work. Subject 3 showed an increase in all outcome variables, including self-selected speed, while subject 4 showed decreases in all variables. These changes may hold insight into a subject’s adoption to a new prosthesis. For instance, a user who exhibits a decrease in prosthetic limb work and speed (subject 4) may indicate a rejection or nonresponse to a new device. In other individuals, a user could increase speed, commonly seen as a beneficial outcome, but not increase prosthetic limb work. This result may mean a patient is depending too much on their sound limb for locomotion, further increasing gait asymmetry. It is important to view the individual’s walking speed in the context of limb work distribution, as only observing gait speed could lead a patient to continue overusing their sound limb, creating complications in the future.

In this study, the participants were not necessarily trained or instructed on how to maximize prosthetic energy return; yet most participants (14 out of 22 participants) chose to walk in a way that increased prosthetic positive work and self-selected speed. In the future, it may be interesting to see whether targeted interventions, such as using real-time propulsive force feedback [38], could further engage individuals to utilize the spring-like function of ESR prostheses. Also, the correlation between prosthetic energy return and walking speed (Fig. 4a) may indicate that interventions that encourage fast walking could be another way to enhance prosthesis mechanics.

The current study is not without limitations. As stated previously, speed-matched trials were not performed post-adaptation, however this has led to a deeper level of analysis than previously anticipated. Some subjects’ speed changes could be below previously-reported minimum detectable thresholds for walking velocity [39]. However, we believe the concepts developed in the current study still hold merit and are applicable to the subjects exhibiting dramatic changes in walking speed. The mechanical work analysis performed in this study can reveal how much energy a prosthesis can store and return; yet, such analysis may not identify how the energy is being transferred from the user to the prosthesis, and vice versa. Additional analyses, such as a power flow analysis [40, 41] may be better equipped to understand how mechanical energy flows throughout the body, and could further elucidate the interaction between the user and the prosthesis. In addition, it should be mentioned that the subjects appearing in the current study are all experienced prosthesis users, with an average of 7.5 years post-amputation. As such, drawing conclusions to initial gait retraining of people with amputations is limited. Lastly, it is currently unclear whether 3 weeks is sufficient to allow individuals to fully adapt to a new prosthesis. We felt that 3 weeks was appropriate based on recommendation from English et al. [42], and in light of the study design which included exchanging only one aspect/component of the prosthesis rather than the entire prosthesis setup (i.e., socket and suspension system remained unchanged). It is possible that a longer adaptation time could result in further changes in gait outcomes, but we opted to keep the adaptation period to 3 weeks to minimize potential drop-outs from the participants.

## Conclusions

After 3 weeks of adaptation to a new prosthetic device, users exhibited an increase in positive prosthesis and sound ankle-foot work, as well as an increase in self-selected speed. Increased prosthesis work could reflect a greater step-by-step utilization of a prosthesis’ spring-like property, suggesting that prosthesis users can change their gait mechanics to better interact with their prosthesis. Analyzing mechanical work profiles may be desirable for tracking gait rehabilitation or response to a prosthesis over time. Future directions for this work could entail analyzing new prosthesis users to fully understand the process of prosthesis adaptation, correlating mechanics-based measures to patient-reported outcomes (such as comfort, ease of use, appearance, and sound), and determining whether targeted interventions could further increase prosthetic energy utilization.

## Additional file

Additional file 1: Ankle-foot work ratio (positive/negative work), stride length, and stance time were examined during the 3 week adaptation period. One factor repeated measures ANOVA was used to examine the effects of visits on the outcome variables. *denotes statistical significance (*p* < 0.05). (PDF 201 kb)
