# Supplementary material for: Prosthetic energy return during walking increases after 3 weeks of adaptation to a new device
Source: J Neuroeng Rehabil. 2018 Jan 27;15:6. doi: 10.1186/s12984-018-0347-1 (PMC5787280; doi:10.1186/s12984-018-0347-1)
Supplement: Additional file 1: — Ankle-foot work ratio (positive/negative work), stride length, and stance time were examined during the 3 week adaptation period. One factor repeated measures ANOVA was used to examine the effects of visits on the outcome variables. *denotes statistical significance (p < 0.05). (PDF 201 kb) [file 12984_2018_347_MOESM1_ESM.pdf]

		Week 0	Week 1.5	Week 3	p-value
**Ankle-Foot Work Ratio**	**Prosthesis**	0.482 ± 0.091	0.493 ± 0.093	0.495 ± 0.081	0.193
**(positive work/negaive work)**	**Sound**	0.732 ± 0.293	0.722 ± 0.264	0.745 ± 0.291	0.612
**Stride Length (m)**	**Prosthesis**	1.333 ± 0.232	1.358 ± 0.226	1.357 ± 0.222	0.362
	**Sound**	1.321 ± 0.207	1.346 ± 0.221	1.373 ± 0.223	*0.024
**Stance time (seconds)**	**Prosthesis**	0.750 ± 0.079	0.731 ± 0.071	0.732 ± 0.072	*0.029
	**Sound**	0.753 ± 0.079	0.736 ± 0.075	0.736 ± 0.074	0.059

Additional file 1: Ankle-foot work ratio (positive/negative work), stride length, and stance time were examined during the 3 week adaptation period. One factor repeated measures ANOVA was used to examine the effects of visits on the outcome variables. *denotes statistical significance ($p < 0.05$)
